# Chromophore pre-maturation for improved speed and sensitivity of split-GFP monitoring of protein secretion

**DOI:** 10.1038/s41598-018-36559-x

**Published:** 2019-01-22

**Authors:** Magnus Lundqvist, Niklas Thalén, Anna-Luisa Volk, Henning Gram Hansen, Eric von Otter, Per-Åke Nygren, Mathias Uhlen, Johan Rockberg

**Affiliations:** 1 KTH - Royal Institute of Technology, School of Chemistry, Biotechnology and Health, Department of Protein Science, Stockholm, Sweden; 2 The Novo Nordisk Foundation Center for Biosustainability, Technical University of Denmark, Kongens Lyngby, Denmark; 3 School of Biological Sciences, Nanyang Technological University, 637551 Singapore, Singapore; 4 KTH - Royal Institute of Technology, Science for Life Laboratory, Stockholm, Sweden

## Abstract

Complementation-dependent fluorescence is a powerful way to study co-localization or interactions between biomolecules. A split-GFP variant, involving the self-associating GFP 1–10 and GFP 11, has previously provided a convenient approach to measure recombinant protein titers in cell supernatants. A limitation of this approach is the slow chromophore formation after complementation. Here, we alleviate this lag in signal generation by allowing the GFP 1–10 chromophore to mature on a solid support containing GFP 11 before applying GFP 1–10 in analyses. The pre-maturated GFP 1–10 provided up to 150-fold faster signal generation compared to the non-maturated version. Moreover, pre-maturated GFP 1–10 significantly improved the ability of discriminating between Chinese hamster ovary (CHO) cell lines secreting GFP 11-tagged erythropoietin protein at varying rates. Its improved kinetics make the pre-maturated GFP 1–10 a suitable reporter molecule for cell biology research in general, especially for ranking individual cell lines based on secretion rates of recombinant proteins.

## Introduction

Biologicals and therapeutic proteins in particular are rapidly increasing their share of the pharmaceutical market. Due to an increasing global demand, improvements of cell lines and bioprocess methodology are highly needed to enhance production capabilities^1–3^. Chinese hamster ovary (CHO) cells are usually the preferred host for production of therapeutic glycoproteins, as these cells are able to provide high product yields and perform post-translational processing, in particular glycosylation, similar to human counterparts^4^. The development of a stable cell line for large-scale production typically involves labor-intensive screening of cells from a polyclonal cell pool. This comprises many cultivation steps of individual candidate clones where the product titer is assessed by, for example, enzyme-linked immunosorbent assays (ELISA)^5^.

As a convenient alternative to ELISA, we have previously presented a homogenous assay to quantify secreted proteins using a split-GFP reporter system^6^. GFP can be genetically divided into subunits which in practice are non-fluorescent until they self-associate. In one such system, GFP is split into two parts: the first part comprises the first ten beta sheets of GFP (GFP 1–10 OPT, henceforth referred to as GFP 1–10) while the second part consists of the eleventh sheet (GFP 11)^7^. GFP 1–10 carries the amino acid residues that make up the non-mature framework of the GFP chromophore. The chromophore becomes maturated upon complementation with the GFP 11 peptide, which can be genetically fused to a protein of interest. This setup has been used for different applications, e.g., solubility assays^8^, studies of protein aggregation^9^ and cellular localization^10^. In our previously described method for split-GFP based protein quantification^6^, GFP 11 is genetically fused to a recombinant protein destined for secretion. As the protein is secreted into the cell supernatant, it can be fluorescently detected and quantified by addition of GFP 1–10. This approach does not require any antibodies or capture/washing steps and the GFP 11 tag is small and has been engineered to minimize perturbation on fusion protein folding and solubility^7^. This split-GFP approach for measuring secreted protein titers could also allow for integration with droplet microfluidics for high-throughput screening of live single cells^11^. This can facilitate the process of finding high-producing clones for therapeutic proteins but also implies a relatively complex environment in which detection limits and assay speed become crucial factors. While the binding event between GFP 1–10 and GFP 11 is relatively fast, the subsequent maturation of the GFP chromophore requires approximately six hours^7,12^, where an autocatalytic cyclization of the three residues which form the chromophore is required^13^.

The purpose of this work is to investigate if an increased sensitivity and accelerated detection in *in vitro* split-GFP assays could allow for a more reliable way to distinguish between protein secretion rates of different cell lines. In a previous study, examining the association and dissociation of split-GFP subunits, it was shown that fluorescent and thus maturated complexes between GFP 1–10 and GFP 11 could be disassembled and then reassembled and still have the ability to fluoresce^14^. Different methods has thereafter been used to generate prematurated versions of split-GFP either by producing GFP with an incorporated proteolytic site that allows for the release of a beta sheet^15^ or by co-expressing a fusion protein with the missing GFP strand^16^. These prematurated split-GFP versions have been seen to provide a fluorescent signal more quickly upon complementation compared to non-maturated variants.

Here, we describe a new protocol for production of GFP 1–10 proteins with maturated chromophores (henceforth referred to as GFP 1–10^mat^) with the aim to simplify existing protocols, which require more purification steps. This is accomplished via capture of inclusion body purified GFP 1–10 on a solid support containing the GFP 11 partner, followed by acid elution of GFP 1–10^mat^ and protein refolding. Further, we demonstrate the advantages of using GFP 1–10^mat^ in complementation assays involving quantification of GFP 11-tagged recombinant erythropoietin (EPO) secreted from CHO cells.

## Results and Discussion

**Figure 1:**
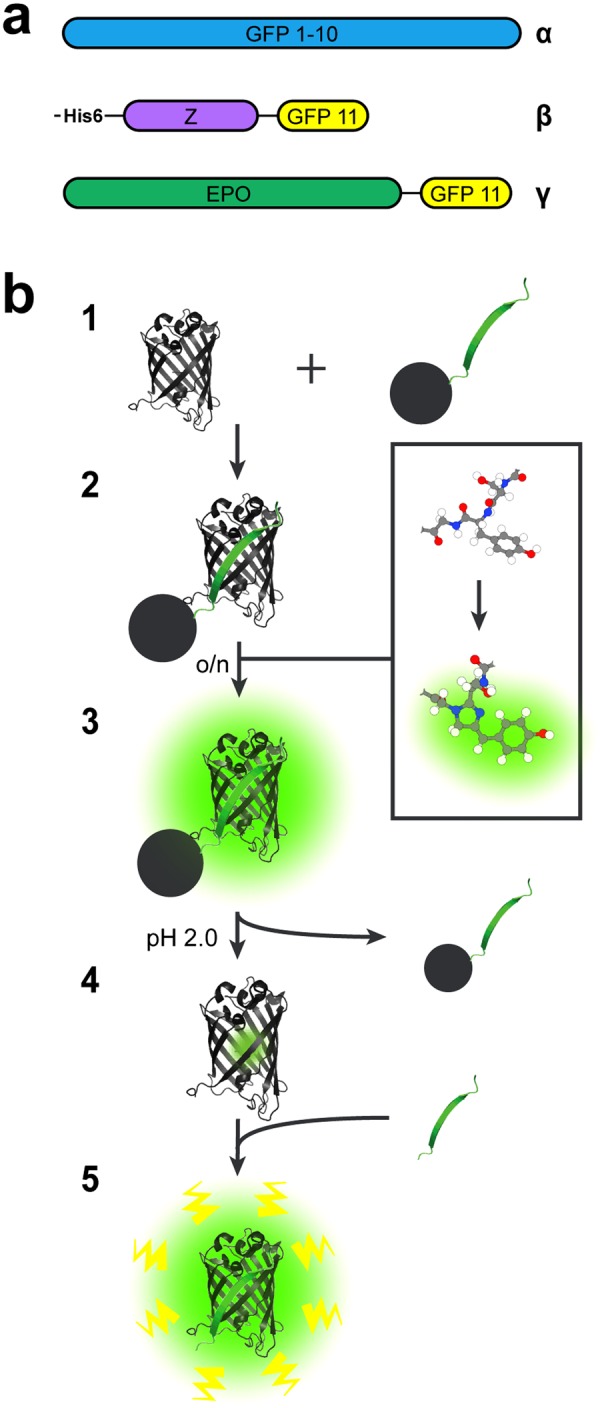
Recombinant proteins used in this study and GFP 1–10 pre-maturation scheme. (**a**) Schematic representation of the domains present in the proteins used in this study: (α) GFP 1–10 (β) His_6_-Z_GFP 11, and (γ) EPO_GFP 11. Both His_6_-Z_GFP 11 and EPO_GFP 11 have a short GS-linkers before GFP 11. (**b**) Illustration of the GFP 1–10 pre-maturation process on a solid support containing His_6_-Z_GFP 11. (1) The 11th sheet of GFP fused to the Z domain derived from protein A (His_6_-Z_GFP 11) is covalently coupled to Sepharose beads. The remaining part of GFP (GFP 1–10) is supplemented to the beads, (2) whereupon the two subunits self-associate and thereby complete the GFP structure. This initiates the relatively slow rearrangement of covalent bonds, which is necessary for the chromophore present in GFP 1–10 to mature. (3) Eventually, GFP 1–10 bound to the beads via GFP 11 will start to fluoresce as their chromophores have been maturated. Unbound GFP 1–10 and other impurities are washed away (**4**) before the bound GFP 1–10^mat^ is released from GFP 11 by low pH and the eluate is neutralized. (**5**) The released GFP 1–10^mat^ now comprises a maturated chromophore and is therefore prone to start fluorescing more rapidly when re-encountering GFP 11.

GFP 1–10 and His_6_-Z_GFP 11 (GFP 11 genetically fused to the C-terminus of the IgG binding Z domain derived from protein A) (Fig. 1a) were expressed intracellularly in *E. coli*. The GFP 1–10 protein was purified from solubilized inclusion bodies and the His_6_-Z_GFP 11 protein was purified from the soluble fraction by immobilized metal ion affinity chromatography (IMAC) (see Supplementary Fig. S1 for SDS-PAGE gel analysis of purified proteins).

His_6_-Z_GFP 11 was covalently coupled to N-hydroxysuccinimide (NHS) activated Sepharose beads (2.5 ml of His_6_-Z_GFP 11 at a concentration of 0.87 mM was added to 5 ml of bead slurry). His_6_-Z_GFP 11 beads were subsequently incubated overnight with a GFP 1–10 solution (40 ml at a concentration of 50 µM) to promote the maturation of the GFP 1–10 chromophore. The following day the bead slurry had turned green (data not shown) and after washing the GFP 11-bound GFP 1–10^mat^ was eluted by low pH after which the solution pH was adjusted to neutral (Fig. 1b).

In order to establish a robust protocol for GFP 1–10 chromophore pre-maturation on beads, various parameters were optimized. Both time and temperature for the incubation with His_6_-Z_GFP 11-beads were investigated and an overnight incubation at room temperature was found to be the most advantageous option (Supplementary Fig. S2). In parallel, it was observed that the pre-maturation process did not seem to be light sensitive, as concealing the samples with aluminum foil during chromophore pre-maturation did not show any improvements in subsequent assays (Supplementary Fig. S3). As the His_6_-Z_GFP 11-to-bead ratio increased, the specific yield of GFP 1–10^mat^ (based on obtained fluorescence per mg of His_6_-Z-GFP 11 on beads) decreased. (Supplementary Fig. S4). Still, it was decided to use the highest amount tested of His_6_-Z_GFP 11 (25 mg; 2.5 ml of a concentration of 0.87 mM) to 5 ml of bead slurry, as this generated more GFP 1–10^mat^ per preparation. When using these coupling conditions, essentially all His_6_-Z_GFP 11 was immobilized on the beads (Supplementary Fig. S5). Higher His_6_-Z_GFP 11-to-bead ratios were not tested, as the bead manufacturer did not recommend it. We reused beads up to two times and did not see any apparent loss in efficiency (data not shown). Studying bead recycling and storage time of beads more thoroughly could be valuable if larger quantities of GFP 1–10^mat^ are to be made repeatedly.

Supplying 50 mg of GFP 1–10 (40 ml at a concentration of 50 µM) to 5 ml His_6_-Z_GFP 11-immobilized bead slurry resulted in a yield of 1 mg GFP 1–10^mat^ in the eluate. Thus, only 2% of the supplied GFP 1–10 was maturated and recovered from the beads. This low yield of eluted GFP 1–10^mat^ is presumably due to sterical hindrance on the beads (Supplementary Fig. S4) and GFP 1–10′s that are not functional due to improper refolding after inclusion body purification. Different approaches were evaluated to improve the yield. Urea was tested for GFP 1–10^mat^ bead elution but provided even lower yields and did not show an enhance detection speed compared to non-maturated GFP 1–10. A slight increase in yield was achieved (with guanidine) when using a Pierce^TM^ Protein Refolding Kit but not enough to deviate from the established protocol for GFP 1–10 refolding (data not shown). Higher yields can be achieved with the proteolytic cleavage method though this involves many more purification steps^15^. Nevertheless, in this study, 0.5 µg to 1.5 µg of GFP 1–10^mat^ was typically used for each sample in 96-well plates. Thus, one batch of GFP 1–10^mat^ would still be enough for 650 to 2000 wells.

**Figure 2:**
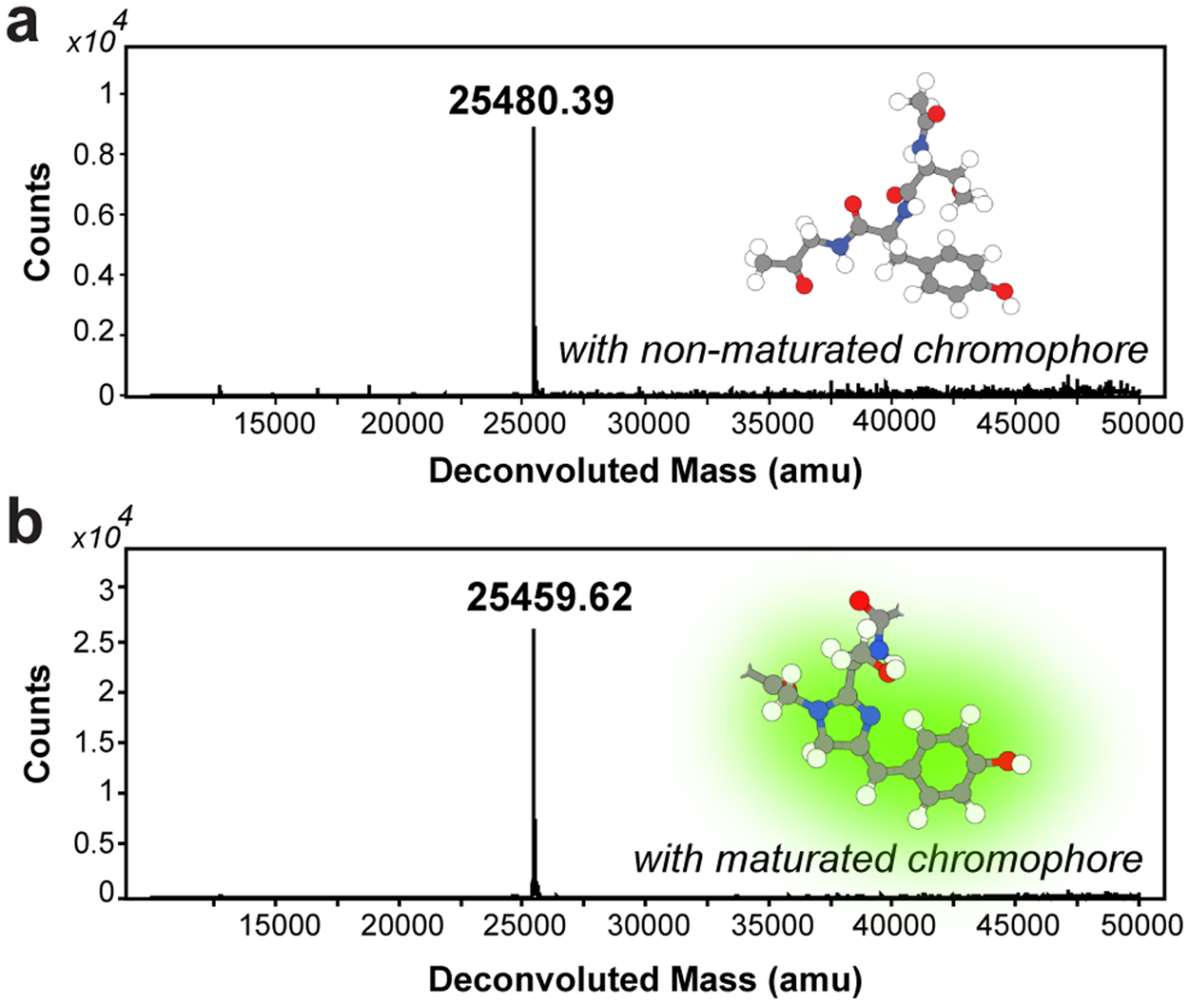
Confirmation of chromophore maturation by mass spectrometry. A comparison of GFP 1–10 before (**a**) and after (**b**) maturation according to the protocol shows a 21 Da (20.77) reduction in mass (white = hydrogen, grey = carbon, blue = nitrogen and red = oxygen). This corresponds to the loss of one oxygen and five hydrogen atoms resulting from the cyclization and covalent rearrangement during the chromophore maturation process.

Chromophore formation in GFP implies a weight loss of 20 or 21 Da, corresponding to a loss of one oxygen and four or five hydrogen atoms depending on maturation theory^13^. Successful maturation was confirmed by detecting this anticipated mass shift (−20.77 Da) by mass spectrometry (Fig. 2), which indicates a loss of five, and not four, hydrogen atoms. In addition, the appearance of only one peak in the MS analysis suggests that all GFP 11-bound and later eluted GFP 1–10^mat^ protein has undergone the maturation during the overnight incubation.

**Figure 3:**
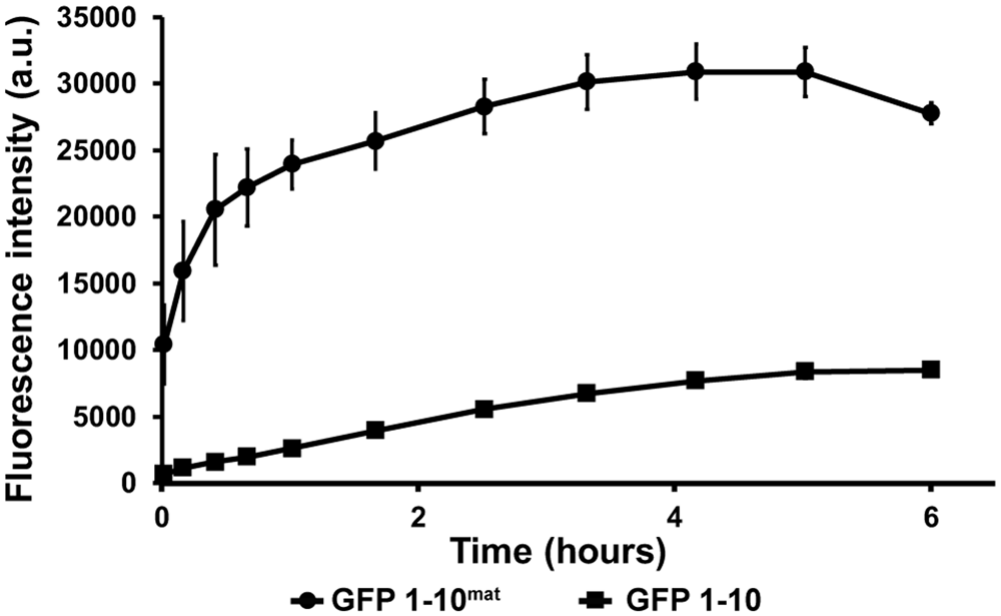
Comparison of the kinetics of fluorescent signal generation using GFP 1–10^mat^ and GFP 1–10. GFP 1–10^mat^ and GFP 1–10 were compared head-to-head in TNG buffer containing His_6_-Z_-GFP 11. His_6_-Z_GFP 11 was excluded in negative controls and the fluorescence from these controls were subtracted from the actual samples. Supplementary Fig. S7 presents a plot with raw data for samples and negative controls. After only two minutes (the first datapoint), the signal from GFP 1–10^mat^ had increased by more than 10,000 a.u. (83 a.u./sec) while the GFP 1–10 steadily increased with only 0.5 a.u./sec until its maximum., thereby making the mature version up to 150-fold faster. Furthermore, the GFP 1–10^mat^ presented a final signal almost four times greater than observed for the GFP 1–10.

The kinetics of complementation-dependent fluorescence of GFP 1–10^mat^ and GFP 1–10 were compared head-to-head in Tris-NaCl-Glycerol (TNG) buffer by monitoring the increase in fluorescent signal after adding His_6_-Z_GFP 11 (Fig. 3). Samples contained 225 µM His_6_-Z_GFP 11 and 0.2 µM of either GFP 1–10^mat^ or GFP 1–10, hence more than a 1000-fold molar excess of His_6_-Z_GFP 11. The signal for GFP 1–10 increased steadily with approximately 0.5 a.u./second until it reached a plateau after ca. five hours, while for GFP 1–10^mat^, the fluorescent signal increased with 83 a.u./sec during the first two minutes after which the signal continued to increase at approx. 6 a.u./sec for an additional hour and later reached a plateau after three hours. Thus, the initial signal formation rate was approx. 150 times faster for the GFP 1–10^mat^ than its non-maturated counterpart. Presumably, the slower increase could be the result of a decline in GFP 1–10^mat^ availability or oligomeric complexes^17^.

When assayed at the same concentration as determined by absorbance at 280 nm and by SDS-PAGE (Supplementary Fig. S6), GFP 1–10^mat^ reached a final signal almost four times as high as seen for GFP 1–10. Notably, compared to the preparation of GFP 1–10 from inclusion bodies, the maturation protocol involves an additional purification step via the GFP 11 during which only the fraction of GFP 1–10 capable of interacting with the bead bound GFP 11 is selectively recovered and other impurities are removed. Interestingly, GFP 1–10^mat^ gave rise to an approximately 250-fold higher background signal (40,000 a.u. and 150 a.u. for GFP 1–10^mat^ and GFP 1–10, respectively). Hence, the eleventh sheet of GFP is not necessary for fluorescence once the chromophore is formed although it enhanced the fluorescence intensity by 3–4 fold (Fig. 3). Higher fluorescence has been described before for prematurated GFP 1–10^15,16^; still, it prompted us to check for co-elution or leakage of His_6_-Z_GFP 11 from the beads used during maturation. However, no such remains were detected by Western blot (Supplementary Fig. S8). The increase in background signal did however not cause any constraints for the assays in this study.

We then examined the emission and excitation spectra of both GFP 1–10 variants (Supplementary Fig. S9). Without His_6_-Z_GFP 11, non-maturated GFP 1–10 did not fluoresce, while GFP 1–10^mat^ showed an excitation plateau around 460–490 nm. Upon addition of His_6_-Z_GFP 11, both GFP 1–10 variants showed similar spectra with a peak at 488 nm. Furthermore, GFP 1–10^mat^ with His_6_-Z_GFP 11 had an increased signal over the entire spectra compared to the background signal of GFP 1–10^mat^ without His_6_-Z_GFP 11, but in particular around the excitation peak at 488 nm.

**Figure 4:**
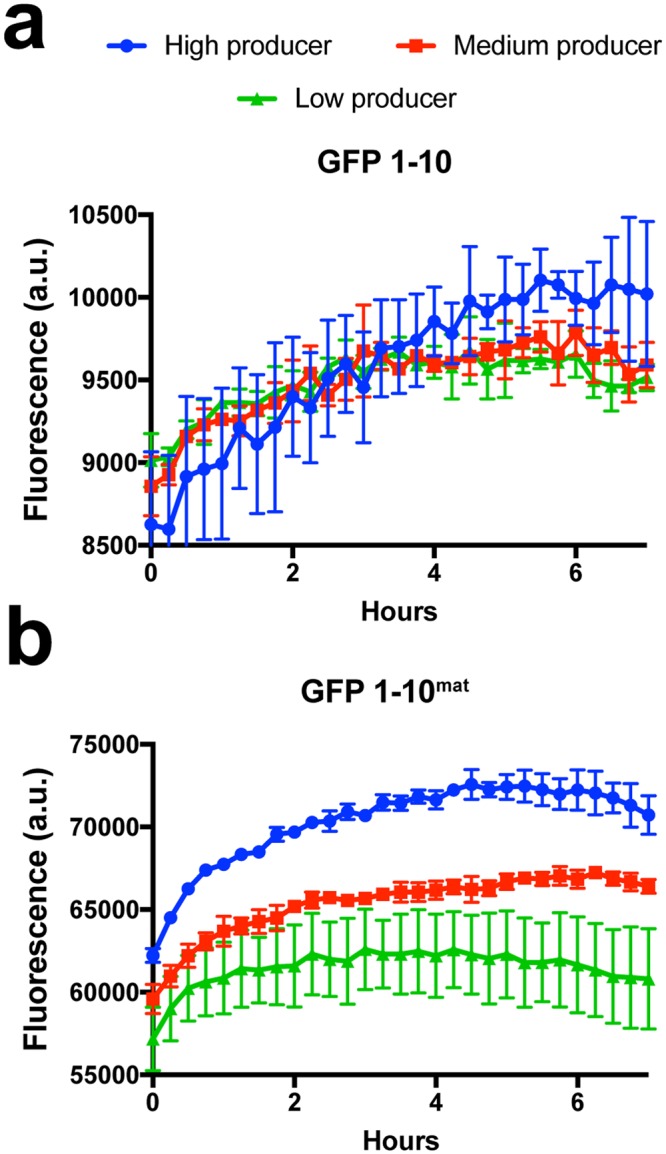
GFP 1–10^mat^ was superior to non-maturated GFP 1–10 for distinguishing between recombinant EPO secretion from CHO cells. CHO cell lines expressing recombinant EPO, genetically tagged with GFP 11, were cultured in microtiter plates with either GFP 1–10^mat^ or GFP 1–10 protein present in the medium from the start and expression was monitored over time. Secreted EPO-GFP 11 resulted in GFP 1–10 complementation and thereby a fluorescent signal. Three strains with different specific productivities were monitored: low producer (“efficiency too low to measure”); medium producer (0.2 pg/cell/day); and high producer (1.5 pg/cell/day). Cultures were run in triplicates and signal values analyzed with a two-tailed, unpaired t-test. To be qualified as differentiable at a certain time point, a p-value below 0.05 was required also for all following time points. (**a**) Using GFP 1–10, it was not possible to discriminate between the cell lines. (**b**) Using GFP 1–10^mat^, it was already from the first time-point possible to distinguish between the high and medium producers, and after 5 hours, it was likewise possible to distinguish the signal between the medium and low producing strains.

To test GFP 1–10^mat^ in a more complex setup, three stable CHO cell lines with different specific productivities of recombinant EPO genetically fused to GFP 11 (EPO_GFP 11) were cultivated in a 96-well plate with either GFP 1–10^mat^ or GFP 1–10 present in the culture medium from the start (Fig. 4). The specific productivities of the low, medium and high producer cell lines had previously been determined to be “below limit of detection”, 0.2 pg/cell/day and 1.5 pg/cell/day, respectively. With GFP 1–10 present in the culture medium, a statistically reliable separation of the productivities between the cell lines was not possible at any timepoint. However, with GFP 1–10^mat^ present, a clear and statistically distinct separation of the productivities could immediately be observed between the high and medium producer cell lines, while the medium and low producer could be differentiated after five hours.

It would be interesting to use our methodology also on other split versions of fluorescent proteins, for example split-mNeonGreen21–10_/11_^18^, which could provide a brighter fluorescent signal *in vitro* compared to GFP 1–10. Other circulated permuted variants of GFP could be tested^16,19^. Another possible route for developing a faster split GFP system is to develop a split version of TurboGFP as it has an inherent rapid maturation^20^ which may allow its use in product titer assays without a pre-maturation step. However, establishing a split version of TurboGFP might be precarious as TurboGFP is more prone to aggregation compared to superfolder GFP, which was used as starting-point for the GFP 1–10 and GFP 11 fragments in this study.

In summary, our strategy to subject GFP 1–10 to a combined pre-maturation and purification step on GFP 11 beads gave rise to up to a 150-fold faster rate of fluorescence complementation compared to its non-maturated counterpart. Moreover, less GFP 1–10^mat^ protein was needed to reach fluorescent signals distinguishable over background. Additionally, the GFP 1–10^mat^ variant made it possible to discriminate between CHO cell lines with different specific productivities of EPO_GFP 11 by monitoring fluorescence in the cell supernatant in real-time.

In conclusion, we recommend the use of GFP 1–10^mat^ for studies where a faster detection and/or improved sensitivity is needed. We especially envision it to be beneficial for sorting of live cells in droplet microfluidics settings based on protein secretion as it allows for significantly shorter incubation times.

## Materials and Methods

### GFP 1–10 and His_6_-Z_GFP 11 production

The gene encoding the GFP 1–10 construct used in the experiments was ordered from DNA2.0 (ATUM) and corresponds to the GFP 1–10 OPT (in the manuscript simply referred to as GFP 1–10) sequence described earlier^7^. The GFP 1–10 gene was expressed in *E. coli* BL21 (DE3) cells using the PJExpress411-Kan vector (ATUM) based on the T7 promoter, and was purified from cell pellets from 500 ml cultivations in accordance with Cabantous *et al*.^21^ although 150 mg of pellets were made instead of 75 mg. His-tagged Z_GFP 11 was expressed in *E. coli* BL21 cells. 500 µl of overnight culture was used to inoculate 500 ml of TSB + Y media with ampicillin (0.1 mg/ml). Cells were maintained at 37 °C and 150 rpm, induced with IPTG (final concentration 1 mM) at OD600 = 1 and harvested the following day. Cells were spun down at 15,000 g for 20 minutes and pellet was stored at −20 °C. Pellet was thawed in 2.5 ml BugBuster/g wet cell weight, vortexed and incubated at room temperature for 20 minutes before spun down 15,000 for 20 minutes. Supernatant containing His_6_-Z_GFP 11 was filtered (0.45 µm) and purified on an ÄKTA start (GE Healthcare) using a HiTrap Nickel FF (cv 1 ml). Protein was eluted in 1 ml fractions using a 0–500 nM imidazole gradient. Eluted protein was buffer exchanged to coupling buffer (0.2 M NaHCO3, 5.5 M NaCl, pH 8,3) on a PD-10 column (GE Healthcare). Protein concentrations were determined by absorbance at 280 nm on an Eppendorf BioPhotometer (Eppendorf). See Supplementary Table T1 for amino acid sequences.

### Bead coupling

His_6_-Z_GFP 11 was covalently coupled to NHS-activated Sepharose beads (GE Healthcare). 5 ml of beads were washed 5 times with 10 ml HCl (1 mM), before His_6_-Z_GFP 11 in coupling buffer was added (0.87 mM, 2.5 ml). Coupling reaction proceeded for 2 hours at room temperature on a rolling mixer before blocking buffer (0.1 M Tris-HCl pH 8.5) was added. Blocking occurred for 2.5 hours on a rolling table. Beads were washed with 10 ml high pH buffer (0.1 M Tris-HCl pH8.5) and then 10 ml low pH buffer (0.1 M acetate buffer, 0.5 M NaCl pH 4.0). This wash was repeated five times. Finally, beads were stored in PBS with 20% ethanol.

### GFP 1–10 pre-maturation

Approximately 50 mg of purified GFP 1–10 in 40 ml TNG buffer (50 mM Tris, pH 7.4, 0.1 M NaCl, and 10% glycerol) was added to 4 ml of coupled beads in a 50 ml Falcon tube and left at room temperature on a rolling table overnight. To clear away non-maturated GFP 1–10, beads were spun down at 1000 rcf for four minutes whereupon the supernatant was removed and beads were resuspended in 40 ml TNG buffer. This washing procedure was repeated twice. GFP 1–10^mat^ was released from the beads with 2 ml glycine buffer (0.1 M, pH 2.0), spun down at 1000 rcf for 4 minutes, and supernatant was neutralized with 2 ml Tris buffer (0.5 M, pH 7.8). This procedure was repeated two more times. Neutralized samples were then filtered (0.45 µm) and buffer exchanged on PD-10 column (GE Healthcare) to TNG buffer. Beads have been reused for at least two times with no apparent loss in efficiency. To recycle beads, they were washed three times with 40 ml of the glycine buffer and one time with 40 ml TNG buffer (spun down at 1000 rcf for 4 minutes between the washes). Finally, beads were stored in 1xPBS with 20% ethanol at 4 °C.

### Mass-spectrometry

GFP 1–10^mat^ and GFP 1–10 were buffer exchanged to MilliQ water. Subsequently, 320 µl of GFP 1–10 samples were mixed with 80 µl of 20% acetonitrile (with 0.1% Trifluoroacetic acid) and purified by RP-HPLC on an analytical column (Zorbax 300SB-C18 4.6 × 150 mm, 3.5 µm particle size, Agilent) using a 30 min gradient of 20–50% buffer B (0.1% TFA in ACN) with a flow-rate of 1.0 ml/min before analyzed by liquid chromatography electrospray ionization mass spectrometry (LC-ESI-MS) on a 6520 Accurate-mass Q-TOF LC/MS (Agilent).

### Split-GFP fluorescent complementation assay

The split-GFP complementation assay was carried out in triplicates for both GFP 1–10 variants. Wells were blocked with 5% BSA in TNG buffer for 10 minutes before the samples were added. Each replicate had a total volume of 100 µl TNG buffer with 225 µM His_6_-Z_GFP 11 and 0.2 µM GFP 1–10. His_6_-Z_GFP 11 was added to the wells (5 µl) before GFP 1–10 (95 µl). The first measurement was done after two minutes. In negative controls, His_6_-Z_GFP 11 was excluded and fluorescence from these controls were subtracted from the actual samples. Measurements were performed at 25 °C every 3 minutes on a CLARIOstar® plate reader (BMG Labtech) at 488 ± 8 nm excitation and 535 ± 30 nm emission.

### EPO_GFP 11-producing CHO cell lines

CHO-S cells were cultivated and transfected essentially as previously described using FreeStyle MAX reagent with an EPO-GFP 11-encoding plasmid harboring a neomycin resistance cassette^6^. This plasmid was constructed essentially like previously described^6^. Geneticin-selected cells were single cell-sorted on a BD FACSJazz cell sorter (BD Biosciences, San Jose, CA) and three randomly picked clones were further characterized in 6-well plates. Viable cell density was measured daily using the previously described Celígo-based method^6^. Supernatant (2000 *g*, RT, 5 min) was obtained three days after seeding for EPO titer analysis performed on an Octet RED96 (Pall ForteBio, Fremont, CA, USA) as described before^22^. Integral of viable cell density (IVCD) and daily specific production rate were calculated as described elsewhere^23^.

### Monitoring secretion rate of EPO_GFP 11 by fluorescent complementation

A black 96-well plate (Nunc) was blocked with 0.5% BSA in TNG buffer for 1.5 h. 50 µL of a 1.6*10^7^ cells/mL cell suspension in fresh medium was added to the blocked plate and mixed with 50 µL of either mature or non-mature GFP 1–10 to yield a final concentration of 15 µg/mL 0.6 µM GFP 1–10. The plate was kept in a CLARIOstar® plate reader (BMG Labtech) for 7 h, at 25 °C and atmospheric CO_2_ concentration with constant shaking between the measurements. GFP fluorescence was measured at 25 °C every 15 minutes for 7 hours in a CLARIOstar® plate reader at 488 ± 8 nm excitation and 535 ± 30 nm emission.

## Electronic supplementary material

Supplementary information
