# Supplementary material for: Chromophore pre-maturation for improved speed and sensitivity of split-GFP monitoring of protein secretion
Source: Sci Rep. 2019 Jan 22;9:310. doi: 10.1038/s41598-018-36559-x (PMC6342966; doi:10.1038/s41598-018-36559-x)
Supplement: Supplementary file 1 — Supplementary information [file 41598_2018_36559_MOESM1_ESM.pdf]

Chromophore pre-maturation for improved speed and sensitivity of split-GFP monitoring of protein secretion

Magnus Lundqvist[1#], Niklas Thalén[1#], Anna-Luisa Volk[1], Henning G. Hansen[2], Eric von Otter[3], Per-Åke Nygren[1], Mathias Uhlen[1,2,4] and Johan Rockberg[1*]

[1]KTH - Royal Institute of Technology, School of Chemistry, Biotechnology and Health, Department of Protein Science, Stockholm, Sweden
[2]The Novo Nordisk Foundation Center for Biosustainability, Technical University of Denmark, Kongens Lyngby, Denmark
[3]School of Biological Sciences, Nanyang Technological University, 637551, Singapore
[4]KTH - Royal Institute of Technology, Science for Life Laboratory, Stockholm, Sweden

Supplementary figure S1 - SDS-PAGE gel analysis of purified His_6-Z_GFP 11 and GFP 1-10 proteins

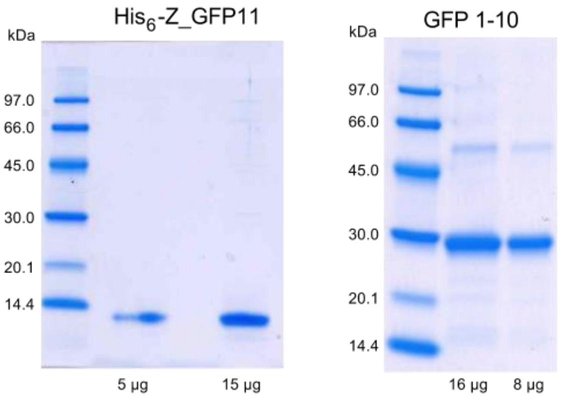

Figure S1. SDS-PAGE analysis of purified His_6-Z_GFP 11 and GFP 1-10 proteins. His_6-Z_GFP 11, with a theoretical molecular weight of 11.9 kDa, was purified by immobilized metal ion affinity chromatography (IMAC) and showed a high purity (left). The GFP 1-10 protein, with a theoretical molecular weight of 25.5 kDa was purified via refolding of inclusion bodies. A band present at 50 kDa could indicate the presence of dimers in the preparation, as described before (Cabantous *et al*., 2005). Both proteins were analysed by SDS-PAGE under reducing conditions using precast BIO-RAD Mini-PROTEAN® TGX™ gels and stained with Thermo Scientific GelCode™ Blue Safe Protein Stain. A low molecular weight ladder from GE healthcare was used for calibration. Protein concentrations were determined by measuring absorbance at 280 nm (A_{280}).

Supplementary figure S2 - Evaluation of incubation temperature and time for GFP 1-10 chromophore pre-maturation on His$_6$-Z_GFP 11-coupled beads

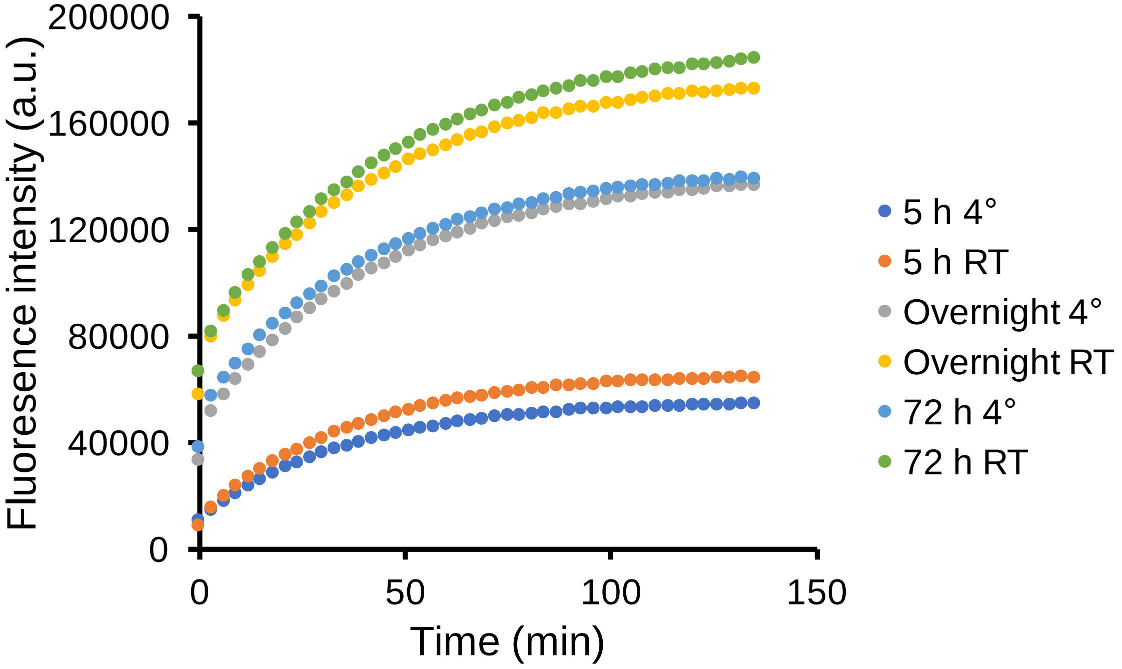

Figure S2. Evaluation of incubation temperature and time for GFP 1-10 chromophore pre-maturation on His$_6$-Z_GFP 11-coupled beads. Approximately 100 µl bead slurry of His$_6$-Z_GFP 11-coupled beads were mixed with 1 mL of 2 mM GFP 1-10 in 1.5 ml tubes. The maturation protocol was carried out for five hours, overnight, or for 72 hours and at 4°C or room temperature as indicated. After acid release and pH adjustment of GFP 1-10^{mat}, His$_6$-Z_GFP 11 protein was added for monitoring of the fluorescence complementation. The highest fluorescence was achieved for maturation for 72 hours at room temperature. However, there was only a slight difference seen between 72 hours and overnight incubation at RT; hence, the quicker overnight protocol was selected for future pre-maturations.

Supplementary figure S3 – No detectable difference between pre-maturation in the absence or presence of light

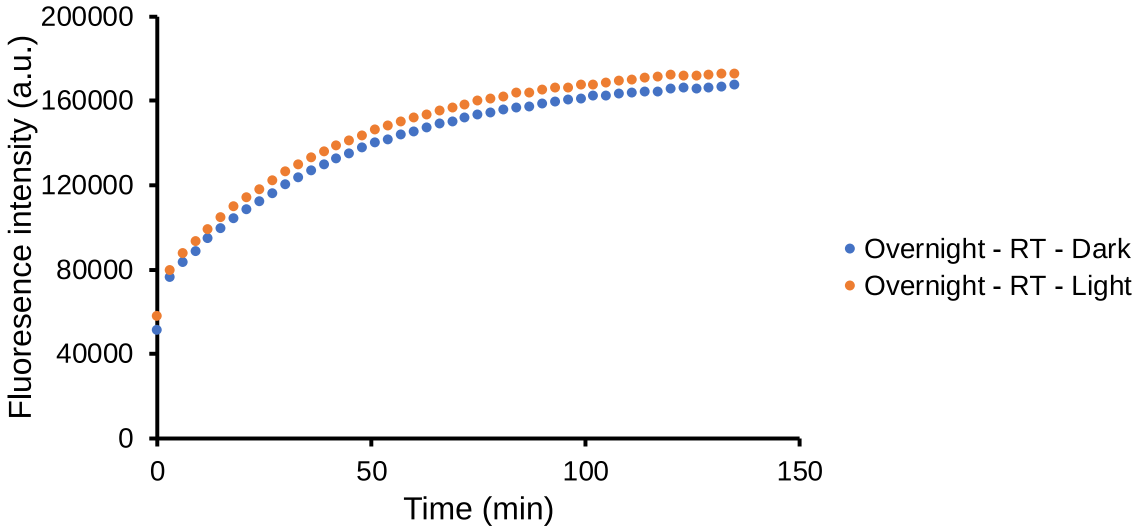

Figure S3. No detectable difference between pre-maturation in the absence or presence of light. Approximately 100 µl bead slurry of His_6-Z_GFP 11-coupled beads were mixed with 1 ml of 2 mM GFP 1-10 in 1.5 ml tubes. The pre-maturation was carried out at room temperature overnight in transparent plastic tubes, either covered in aluminum foil (dark conditions) or left uncovered in illuminated laboratory space (light conditions). His_6-Z_GFP 11 was subsequently added to acid-released and pH-adjusted GFP 1-10^{mat}. No apparent difference in the fluorescence development kinetics was seen between the GFP 1-10^{mat} that had been exposed to light during the pre-maturation from that not exposed to light.

Supplementary figure S4 - Effects of bead-to-His$_6$-Z_GFP 11 ratio on GFP 1-10 pre-maturation

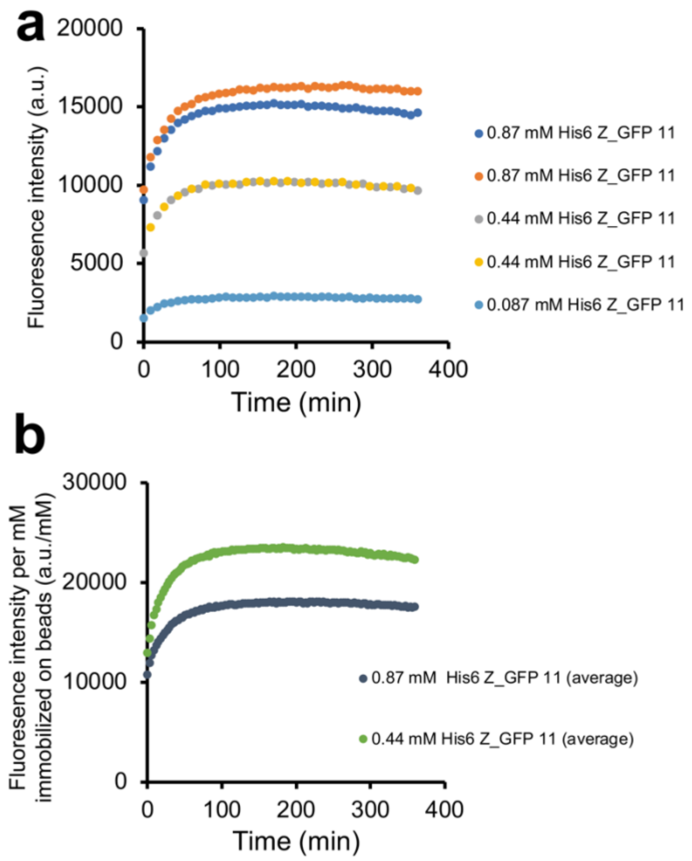

Figure S4. Effects of bead-to-His$_6$-Z_GFP 11 ratio on GFP 1-10 pre-maturation. (a) 2.5 ml of 0.87 mM, 0.44 mM or 0.087 mM solutions of His$_6$-Z_GFP 11 were coupled to 5 ml of NHS-activated bead slurry. 40 ml of 50 µM GFP 1-10 was subsequently added to the beads for pre-maturation overnight at room temperature. After incubation, beads were washed three times in TNG buffer and eluted with 0.1 M glycin (pH 2.0). The eluate was neutralized to pH 7.4 by addition of an equal volume of 0.5 M Tris-HCl buffer (pH 7.8). The buffer was subsequently exchanged to TNG on a PD-10 desalting column. The fluorescence of the GFP 1-10^{mat} was measured in duplicates (one replicate for 0.087 mM sample) after the addition of 5 times molar excess of His$_6$-Z_GFP 11 protein.
The amount of GFP 1-10^{mat} increased with higher densities of His$_6$-Z-GFP 11 on the beads. **(b)** However, a decrease in the specific yield (obtained fluorescence signal per mg of His$_6$-Z-GFP 11 on beads) was seen for beads immobilized with 0.87 mM compared to 0.44 mM, presumably due to sterical hindrance.

Supplementary figure S5 - Coupling of His$_6$-Z-GFP 11 to NHS-activated beads

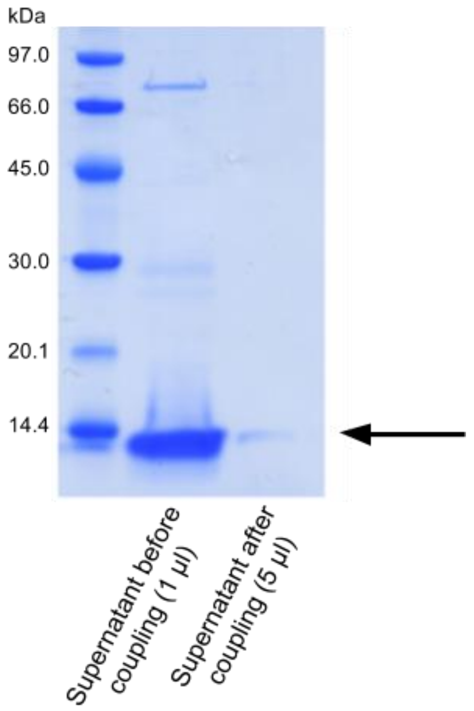

Figure S5. Coupling of His$_6$-Z-GFP 11 to NHS beads. To investigate the capacity of the NHS-activated beads, the highest concentration investigated (2.5 ml of 0.087 mM) of His$_6$-Z-GFP 11 was coupled to 5 ml of NHS-activated bead slurry according to the manufacturer's protocol. The amounts of ligand in the supernatant from before (1 µl sample) and after (5 µl sample) coupling was analyzed by SDS-PAGE and Coomassie staining. The results showed that essentially all of the applied His$_6$-Z-GFP 11 protein had been immobilized on the beads from the 0.87 mM His$_6$-Z-GFP 11 solution.

Supplementary figure S6 - Concentration comparison of GFP 1-10 and GFP 1-10^{mat} by SDS-PAGE

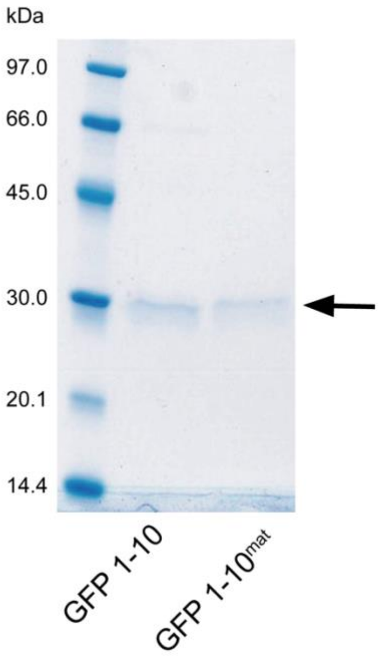

Figure S6. Concentration comparison of GFP 1-10 and GFP 1-10^{mat} by SDS-PAGE. The concentration in solutions of the two versions were compared on a Coomassie-stained SDS-PAGE gel to investigate whether the samples had the same amount of GFP 1-10. The concentration determination by absorbance at 280 nm used to obtain equal amounts for fluorescence assays could potentially have been misleading if GFP 1-10 had more impurities compared to GFP 1-10^{mat}. Hence, The same theoretical amounts (1 µg) of GFP 1-10 and GFP 1-10^{mat} (according to A_{280} concentration determination) were loaded on the SDS-PAGE gel. No clear difference in concentration was seen for the GFP 1-10 solutions used in the complementation assays.

Supplementary figure S7 – Raw data plot for comparison of the kinetics of fluorescent signal generation using GFP 1-10^{mat} and GFP 1-10

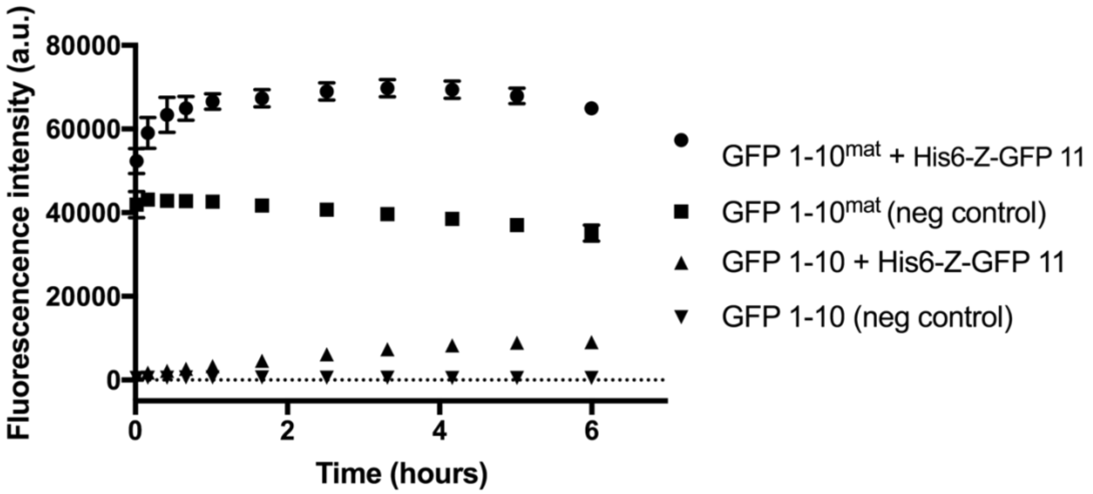

Figure S7: Raw data plot for comparison of the kinetics of fluorescent signal generation using GFP 1-10^{mat} and GFP 1-10. GFP 1-10^{mat} and GFP 1-10 were compared head-to-head in TNG buffer containing His6-Z-GFP 11. This figure presents the same data as Figure 3; here, the negative control have not been subtracted from the actual samples to demonstrate the autofluorescence of GFP 1-10^{mat}. Three replicates were used for both actual samples and negative controls. A plot with only non-maturated GFP 1-10 can be found below.

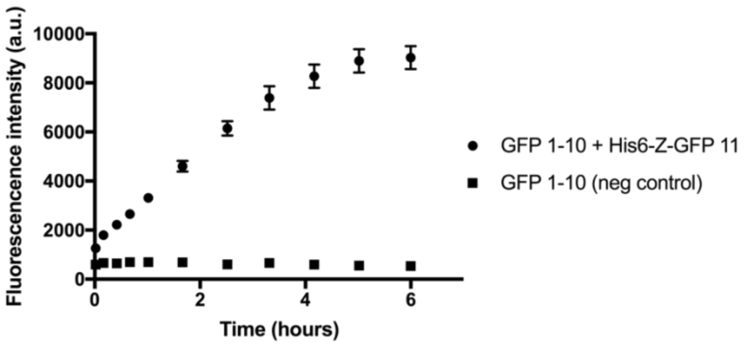

Supplementary figure S8 – No His_6-Z_GFP 11 could be detected by western blotting in the eluted and pre-maturated GFP $1\text{-}10^{mat}$

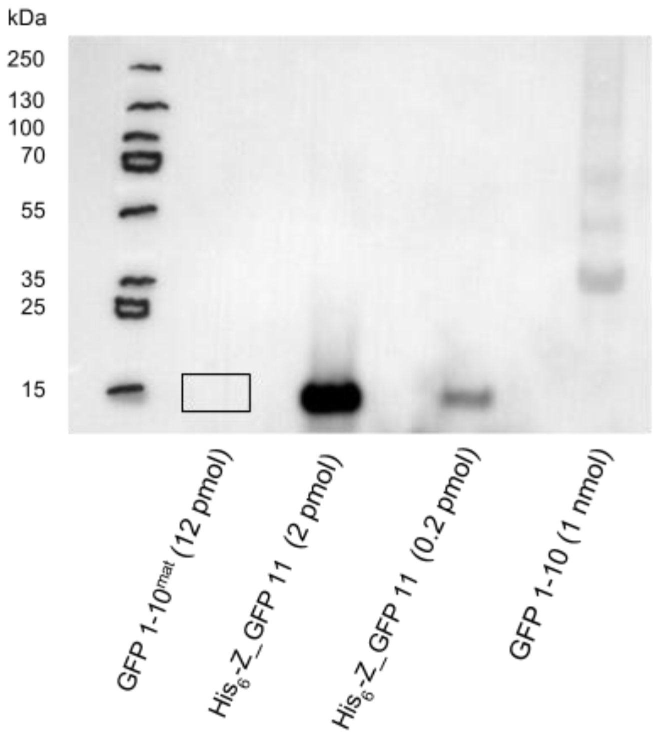

Figure S8: No His_6-Z_GFP 11 protein could be detected by Western blotting in the eluted and pre-maturated GFP $1\text{-}10^{mat}$. To investigate if any traces of His_6-Z-GFP 11 protein could be detected in the eluted and pre-maturated GFP $1\text{-}10^{mat}$, a Western blotting analysis was performed. 15 µl of eluted and pre-maturated GFP $1\text{-}10^{mat}$ (12 pmol), 1 nmol GFP 1-10, 2 pmol His_6-Z-GFP 11 and 0.2 pmol His_6-Z-GFP 11 were separated on a NuPAGE 4-12% Bis-Tris gel and transferred onto an Invitrolon™ (ThermoFisher) membrane. The membrane was incubated for 1 h at room temperature with 1:5000 diluted goat anti-Z (Affibody AB) in blocking buffer. Subsequent to washing, the membrane was incubated with 1:5000 diluted anti-goat peroxidase-conjugated antibody in blocking buffer for 1 h. After the secondary staining, the membrane was washed and developed with 10 ml of Immobilon Chemiluminescent HRP Substrate (Millipore) for 5 min in room temperature and placed in a plastic seal. A marker pen has been used to visualize the ladder. Chemiluminescence was detected with ChemiDoc XRS+ (BIO-RAD) apparatus and Image lab 5.2 software. A 5% (w/v) dry milk and 0.5% Tween 20 solution in PBS was used as blocking solution and 0.1% Tween 20 solution in PBS as wash solution.

No band at 11.9 kDa (see black box in figure), corresponding to Z_GFP 11 could be detected in the GFP $1\text{-}10^{mat}$ preparation. A strong signal could be seen for a 2 pmol sample of Z_GFP 11. Reassuringly, when this amount was used in fluorescence complementation assays, no fluorescence signal could be detected regardless of gain settings or GFP $1\text{-}10^{mat}$ amount (data not shown).

Supplementary figure S9 – Emission and excitation spectra for GFP 1-10 and GFP 1-10^{mat}

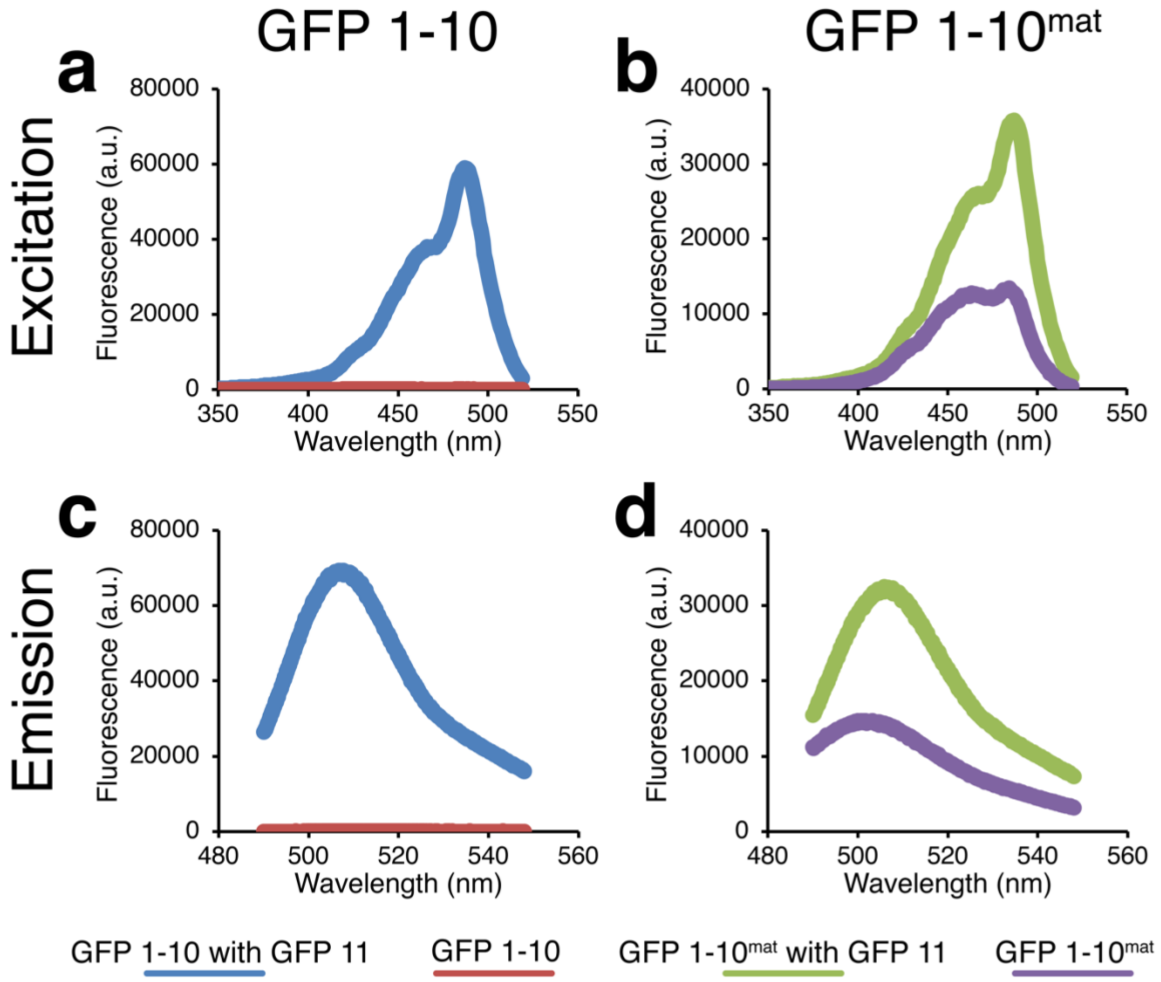

Fig. S9. Emission and excitation spectra for GFP 1-10^{mat} and GFP 1-10. Excitation and emission spectra were measured on a CLARIOstar® plate reader (BMG Labtech) with emission set to 545 nm (±8 nm) for recording of excitation spectra and with excitation set to 466 nm (±8 nm) for recording of emission spectra. Measurements were conducted with i) 4 500 pmol GFP 1-10 and 4700 pmol His$_6$-Z_GFP 11, (ii) 4500 pmol GFP 1-10 alone, (iii) 94 pmol GFP 1-10^{mat} and 4700 pmol His$_6$-Z_GFP 11 and (iv) 94 pmol GFP 1-10^{mat} alone in TNG buffer and a total volume of 100 µl. The large amount of non-maturated GFP 1-10 used in the assays was necessary to get readable signals. Excitation and emission measurements were done at various time points after addition of His$_6$-Z_GFP 11 but the here presented excitation and emission spectra were generated after 90 minutes and 2 hours, respectively. Measurements were also performed at 488 nm excitation and 520 nm emission to evaluate the signal at the maximum emission and excitation wavelengths (data not shown). These results showed the same excitation and emission patterns for both GFP 1-10 and GFP 1-10^{mat}, but due to limitation of overlapping signals, they did not show the full spectral view of interest for each protein. The results showed that the

excitation and emission spectra were the same for GFP 1-10 and GFP 1-10^{mat} when analyzed together with His_6-Z-GFP 11 (blue and green in **A-D**). The excitation and emission peaks for both variants were 488 and 506 nm, respectively. However, even without His_6-Z-GFP 11 present, GFP 1-10^{mat} showed an excitation plateau between 460-490 nm (purple in **B)** and an emission peak at 503 nm (purple in **D**). The excitation and emission for GFP 1-10 without His_6-Z-GFP 11 was negligible (red in **A** and **C**). The ratio between the excitation at 460 and 488 nm for GFP 1-10^{mat} with His_6-Z_GFP 11 present increased over time; it was 1.26 after a few minutes and 1.46 after 90 min. For GFP 1-10 with His_6-Z_GFP 11 present, the ratio was always around 1.5.

Supplementary table T1 - Amino acid sequences

Protein name	Amino acid sequence
GFP 1-10 OPT<br>(referred to as GFP 1-10)	MHHHHHHGSANSSGSS§1KGEELFTGVVPILVELDGDVNGHKFSVRGEGEGDATIGKLTLKFICTTGKLPVPWPTLVTTLTYGVQCFSRYPDHMKRHDFFKSAMPEGYVQERTISFKDDGKYKTRAVVKFEGDTLVNRIELKGTDFKEDGNILGHKLEYNFNSHNVYITADKQKNGIKANFTVRHNVEDGSVQLADHYQQNTPIGDGPVLLPDNHYLSTQTVLSKDPNEK
His_6-Z_GFP 11<br><br>(GFP 11 underlined)	MTMITPSLHHHHHHVDNKFNKEQQNAFYEILHLPNLNEEQRNAFIQSLKDDPSQSANLLAEAKKLNDAQAPKQIQSDPRVPVATGGSGRDHMVLHEYVNAAGITAAA
EPO_GFP 11<br><br>(Signal peptide shown with italic letters and GFP 11 underlined)	*MGVHECPAWLWLLLSLLSLPLGLPVLG*APPRLICDSRVLERYLLEAKEAENITTGCAEHCSLNENITVPDTKVNFYAWKRMEVGQQAVEVWQGLALLSEAVLRGQALLVNSSQPWEPLQLHVDKAVSGLRSLTTLLRALGAQKEAISPPDAASAAPLRTITADTFRKLFRVYSNFLRGKLKLYTGEACRTGDRGGGSGGGSRDHMVLHEYVNAAGIT
